# Commutative avatars of representations of semisimple Lie groups

**DOI:** 10.1073/pnas.2319341121

**Published:** 2024-09-11

**Authors:** Tamás Hausel

**Affiliations:** ^a^Hausel group, Institute of Science and Technology Austria, Klosterneuburg 3400, Austria

**Keywords:** representations of Lie groups, Hitchin integrable system, Higgs field, equivariant cohomology, intersection cohomology

## Abstract

Here we announce the construction and properties of a big commutative subalgebra of the Kirillov algebra attached to a finite dimensional irreducible representation of a complex semisimple Lie group. They are commutative finite flat algebras over the cohomology of the classifying space of the group. They are isomorphic with the equivariant intersection cohomology of affine Schubert varieties, endowing the latter with a new ring structure. Study of the finer aspects of the structure of the big algebras will also furnish the stalks of the intersection cohomology with ring structure, thus ringifying Lusztig’s *q*-weight multiplicity polynomials i.e., certain affine Kazhdan–Lusztig polynomials.

## Significance

Representations of continuous symmetry groups by matrices are fundamental to mathematical models of quantum physics and also to the Langlands program in number theory. Here, we attach a commutative matrix algebra, called big algebra, to a noncommutative irreducible matrix representation of a bounded continuous symmetry group. We show that the geometry of our commutative algebras captures sophisticated information of the representation, for example, its weight multiplicities. We have, and expect more, applications to polynomial identities between quantum numbers of baryon multiplets in particle physics, to mathematical problems related to Higgs fields in quantum physics and also to compatibility with Langlands duality in number theory.

## 1. Kirillov and Medium Algebras

Let G be a connected complex semisimple Lie group with Lie algebra g, which we identify with $g≅g^{*}$ using the Killing form. Let $\mu \in \Lambda^{+}\left(\text{G}\right)$ be a dominant weight, and let $\rho_{\mu }:\text{G}\to \text{GL}\left(V^{\mu }\right)$ and $\varrho_{\mu }:=\mathrm{Lie}\left(\rho_{\mu }\right):g\to gl\left(V^{\mu }\right)≅\text{End}\left(V^{\mu }\right)$ be the corresponding complex highest weight representations of the group and its Lie algebra. Using the natural action of G on the symmetric algebra $S^{*}\left(g\right)$ and on the endomorphism algebra $\text{End}(V^{\mu })$ Kirillov (1) introduced

$$C^{\mu }(g)=C^{\mu }:=(S^{*}(g)⊗\text{End}(V^{\mu }){)}^{\text{G}}≅Maps(g≅g^{*}\to \text{End}(V^{\mu }){)}^{\text{G}}$$

which we call (classical) Kirillov algebra..

Kirillov’s motivation for the introduction of $C^{\mu }$ was to understand weight multiplicities of a maximal torus T⊂G. For example, he proved in (1, Theorem S) that $C^{\mu }$ is commutative if and only if *V*^*μ*^ is weight multiplicity free. This means that for all $\lambda \in \Lambda =\mathrm{Hom}(T,C^{\times })$ the weight space $V_{\lambda }^{\mu }$ is at most one dimensional. We will see below, that the big commutative subalgebras of the Kirillov algebra we will introduce in this paper will induce in *Corollary* 2.2 a graded ring structure on multiplicity spaces.

The Kirillov algebra $C^{\mu }$ is an associative, graded $H_{\text{G}}^{2*}:=S^{*}{\left(g\right)}^{\text{G}}≅\mathbb{C}{\left[g^{*}\right]}^{\text{G}}≅\mathbb{C}{\left[g\right]}^{\text{G}}$-algebra. The grading is induced from the usual grading on $S^{*}\left(g\right)$ and the commutative graded C-algebra $H_{\text{G}}^{2*}$ acts by scalar multiplication.

We fix a principal $sl_{2}$-subalgebra ⟨e,f,h⟩⊂g, so that we get a section of χ:g→g//G, the Kostant section $s:=e+g_{f}\subset g^{\text{reg}}$, in particular s≅g//G. Moreover $s\subset g^{\text{reg}}$ contains only regular elements, i.e., ones with smallest dimensional centralizers, and s intersects every G-orbit of $g^{\text{reg}}$ in exactly one point. Because the codimension of $g\setminus g^{\text{reg}}$ in g is 3 we can identify

[1.1] $$\begin{array}{ll} C^{\mu } & ≅Maps{\left(g^{\text{reg}}\to \text{End}(V^{\mu })\right)}^{\text{G}}\\ & ≅Maps\left(f:s\to \text{End}(V^{\mu })|f(x)\in {\left(\text{End}(V^{\mu })\right)}^{{\text{G}}_{x}}\right). \end{array}$$

We can restrict any subalgebra $A\subset C^{\mu }$ to x∈g to get the finite matrix algebra

[1.2] $$\begin{array}{c} A_{x}:=\left\{f\left(x\right)|f\in A\right\}\subset {\left(\text{End}\left(V^{\mu }\right)\right)}^{{\text{G}}_{x}}. \end{array}$$

We will denote the one-parameter subgroup ${\text{H}}^{z}:C^{\times }\to {\text{G}}_{\mathrm{ad}}=\text{G}/Z\left(\text{G}\right)$ integrating ⟨h⟩⊂g. Then, $\text{Ad}\left({\text{H}}^{z}\right)e=z^{-1}e$ and so the $C^{\times }$-action

[1.3] $$\begin{array}{ccc} {\mathbb{C}}^{\times }\times g & \to & g\\ \;\;(z,x) & \mapsto & z\cdot x:=\text{Ad}({\text{H}}^{z})zx \end{array}$$

on g preserves *e* and $g_{f}$ and thus the Kostant section s, and induces the grading on $C^{\mu }$ in Eq. **1.1**.

The most important element of $C^{\mu }$, called the small operator is given by

[1.4] $$\begin{array}{c} \begin{array}{ccc} M_{1}: & g & \to \text{End}(V^{\mu })\\ & A & \mapsto \varrho_{\mu }\left(A\right) \end{array}. \end{array}$$

More generally we will have an element of the Kirillov algebra from any G-equivariant polynomial map F:=g→g by

[1.5] $$\begin{array}{c} \begin{array}{ccc} M_{F}: & g & \to \text{End}(V^{\mu })\\ & A & \mapsto \varrho_{\mu }\left(F\left(A\right)\right) \end{array}. \end{array}$$

For an invariant polynomial $p\in C{\left[g\right]}^{\text{G}}$ we can define its derivative $dp:g\to g^{*}≅g.$ As dp is automatically G-equivariant we have the operator $M_{\mathrm{dp}}$ from Eq. **1.5**, which we call a medium operator. corresponding to $p\in C{\left[g\right]}^{\text{G}}$. For example, we have the small operator of Eq. **1.4** $M_{1}=M_{d\kappa /2}$, where *κ*, the Killing form, is thought of as a degree 2 invariant polynomial. In general, we will fix a generating set $C{\left[g\right]}^{\text{G}}≅C\left[p_{1},\cdots ,p_{r}\right]$ of homogeneous invariant polynomials $p_{i}\in C{\left[g\right]}^{\text{G}}$ of degree *d*_*i*_, s.t. $d_{1}\leq \cdots \leq d_{r}$, where r=rank(G). Then, we also denote $M_{i}:=M_{dp_{i}}$. We will arrange that $p_{1}=\kappa /2$ so that $M_{1}=M_{dp_{1}}$ is our small operator in Eq. **1.4**. Using these medium operators we define

$$M^{\mu }\left(g\right)=M^{\mu }:={\left\langle 1,M_{1},\cdots ,M_{r}\right\rangle }_{H_{\text{G}}^{2*}}\subset C^{\mu }$$

the medium algebra.

In (1, Theorem M) it is proved that the medium operators are central in $C^{\mu }$. (2, *Theorem* 1.1) and the finite dimensional von Neumann double centralizer theorem imply the following:

Theorem 1.1.

1. *For* x∈s *the restriction Eq.* ***1.2*** *satisfies* $M_{x}^{\mu }=\varrho_{\mu }\left(U\left(g_{x}\right)\right)$.
2. $M^{\mu }=Maps(f:s\to \;\text{End}\left(V^{\mu }\right)|f\left(x\right)\in \varrho_{\mu }\left(U\left(g_{x}\right)\right)\subset$$\text{End}\left(V^{\mu }\right))\subset C^{\mu }$. *In particular,* $M^{\mu }$ *is independent of the choice of generating set of* $C{\left[g\right]}^{\text{G}}$.
3. The medium algebra $M^{\mu }=Z\left(C^{\mu }\right)$ *is the center of the Kirillov algebra.*

### 1.1. Limits of Weight Spaces from Common Eigenspaces of $M^{\mu }$.

Denote the maximal torus $\text{T}={\text{G}}_{h+e}\subset \text{G}$ corresponding to the centralizer of the regular semisimple element *h* + *e*. For dominant weights $\mu ,\lambda \in \Lambda^{+}$ we denote by $V_{\lambda }^{\mu }\subset V^{\mu }$ the *λ*-weight space of T in *V*^*μ*^. Motivated by Kostant’s study (3) of the zero weight space $V_{0}^{\mu }$ Brylinski (4) introduced a filtration

[1.6] $$\begin{array}{c} 0<F_{0}<\cdots <F_{p}<F_{p+1}<\cdots <V_{\lambda }^{\mu } \end{array}$$

called the *Brylinski–Kostant filtration*. It is defined using our regular nilpotent e∈g as

$$F_{p}:=\left\{x\in V_{\lambda }^{\mu }:e^{p+1}x=0\right\}.$$

In turn, Brylinski considers the **e*-limit* of $V_{\lambda }^{\mu }$ as

[1.7] $$\begin{array}{c} {\text{lim}}_{e}V_{\lambda }^{\mu }:=\sum e^{p}\cdot F_{p}\subset V^{\mu }. \end{array}$$

The main result of ref. 4 is that

$$\begin{array}{cc} & \sum_{p}\dim\left(F_{p+1}/F_{p}\right)q^{p}\\ & =q^{-(\lambda ,\rho )}\sum_{k}\dim{\left(\left[\lim_{e}V_{\lambda }^{\mu }\right]\right)}^{h=k}q^{\frac{k}{2}}=m_{\lambda }^{\mu }\left(q\right). \end{array}$$

Here *ρ* is the half-sum of positive roots, (,) is the basic inner product and ${\left[\lim_{e}V_{\lambda }^{\mu }\right]}^{h=k}$ the *k*-eigenspace of *h* acting on $\lim_{e}V_{\lambda }^{\mu }$. While

[1.8] $$\begin{array}{c} m_{\lambda }^{\mu }\left(q\right)=\sum_{w\in W}\epsilon \left(w\right)P_{q}\left(w\left(\mu +\rho \right)-\lambda -\rho \right) \end{array}$$

is Lusztig’s (5) *q*-analogue of weight multiplicity. It is defined using the *q*-analogue of Kostant’s partition function: $\prod_{\alpha \in \Delta_{+}}{\left(1-qe^{\alpha }\right)}^{-1}=\sum_{\pi \in \Lambda }P_{q}\left(\pi \right)e^{\pi },$ where $\Delta_{+}\subset \Lambda$ denotes the set of positive roots.

For $z\in C^{\times }$, using the $C^{\times }$-action Eq. **1.3**, let

$$h_{z}:=e+zh=z\cdot \left(e+h\right)\in g$$

a regular semisimple element. Define also the $C^{\times }$-action on the Grassmannian $\text{Gr}(k,V^{\mu })$ of *k*-planes in *V*^*μ*^ by $z\cdot U:=\rho_{\mu }\left(H^{z}\right)\left(U\right)\in \text{Gr}\left(k,V^{\mu }\right)$ for $U\in \text{Gr}(k,V^{\mu })$. Then, we have the following:

Theorem 1.2. *Let* $\lambda \leq \mu \in \Lambda^{+}$, *that is* *λ* *a dominant weight in* *V*^*μ*^, *then we have*

1. *for* $z\in C^{\times }$ *the subspace* $z\cdot V_{\lambda }^{\mu }\subset V^{\mu }$ *is a weight space for the maximal torus* ${\text{G}}_{h_{z}}$ *and thus a common eigenspace for* $M_{h_{z}}^{\mu }=\varrho_{\mu }\left(U\left(g_{h_{z}}\right)\right)$,
2. $\lim_{e}V_{\lambda }^{\mu }=\;\lim_{z\to 0}z\cdot V_{\lambda }^{\mu }$, *i.e., Brylinski’s limit agrees with an actual limit,*
3. $\lim_{e}V_{\lambda }^{\mu }=\;\lim_{z\to 0}z\cdot V_{\lambda }^{\mu }$ *is an eigenspace of* $M_{e}^{\mu }=\varrho_{\mu }\left(U\left(g_{e}\right)\right)$ *thus* $\lim_{e}V_{\lambda }^{\mu }\subset {\left(V^{\mu }\right)}^{{\text{G}}_{e}}$ *(4, Proposition 2.6),*
4. $\lim_{e}V_{\mu_{\min}}^{\mu }={\left(V^{\mu }\right)}^{{\text{G}}_{e}}$ for $\mu_{\min}$ *the minuscule dominant weight in *V*^*μ*^ [(4, *Corollary* 2.7) for* $\mu_{\min}=0$].

## 2. Definition and Basic Properties of Big Algebras

Replacing the symmetric algebra $S^{*}\left(g\right)$ with the universal enveloping algebra U(g), Kirillov in ref. 1 also introduced

$$Q^{\mu }\left(g\right)=Q^{\mu }:={\left(U\left(g\right)⊗\text{End}\left(V^{\mu }\right)\right)}^{\text{G}}$$

the quantum Kirillov algebra, which is an algebra over the center $Z\left(g\right)=U{\left(g\right)}^{\text{G}}$ of the enveloping algebra. The universal enveloping algebra U(g) has a canonical filtration $F_{0}U\left(g\right)\subset \cdots \subset F_{k}U\left(g\right)\subset F_{k+1}U\left(g\right)\subset \cdots$ such that the associated graded algebra $gr\left(U\left(g\right)\right)≅S^{*}\left(g\right)$. The Rees construction for the filtered algebra R=U(g) then yields the graded C[ħ]-algebra

[2.1] $$\begin{array}{c} R_{\hbar }:={⊕}_{i=0}^{\infty }\hbar^{i}F_{i}R. \end{array}$$

The so-obtained algebra $U_{\hbar }\left(g\right)$ interpolates between $U_{1}\left(g\right)≅U\left(g\right)$ and $U_{0}\left(g\right)≅gr\left(U\left(g\right)\right)≅S^{*}\left(g\right).$ We will also consider the *ħ*-quantum Kirillov algebra

$$Q_{\hbar }^{\mu }\left(g\right)=Q_{\hbar }^{\mu }:={\left(U_{\hbar }\left(g\right)⊗\text{End}\left(V^{\mu }\right)\right)}^{\text{G}},$$

which is naturally a $Z_{\hbar }\left(g\right):=U_{\hbar }{\left(g\right)}^{\text{G}}$-algebra. It interpolates between the quantum and classical Kirillov algebras: $Q_{1}^{\mu }≅Q^{\mu }\left(g\right)$ over $Z_{1}\left(g\right)=Z\left(g\right)$ and $Q_{0}^{\mu }≅C^{\mu }\left(g\right)$ over $S^{*}{\left(g\right)}^{\text{G}}≅Z_{0}\left(g\right)$.

Recall from refs. 6 and 7 and specifically from (8, §8.2) the two-point Gaudin algebra $G\subset Q\left(g\right):={\left(U\left(g\right)⊗U\left(g\right)\right)}^{\text{G}}.$ This is defined as a quotient of the Feigin–Frenkel center (9), and thus it is a commutative subalgebra of the universal quantum Kirillov algebra Q(g). We will also take the Rees construction Eq. **2.1** with respect to the filtration on Q and G coming from the filtration on the first copy of U(g) and denote them $G_{\hbar }\subset Q_{\hbar }$. These are graded C[ħ]-algebras, with $G_{\hbar }$ commutative. For $\mu \in \Lambda^{+}\left(\text{G}\right)$ the image $G_{\hbar }^{\mu }:=\pi^{\mu }\left(G_{\hbar }\right)\subset Q_{\hbar }^{\mu }$ under the projection $\pi^{\mu }:Q_{\hbar }\to Q_{\hbar }^{\mu }$ induced from the projection $U\left(g\right)\to \text{End}\left(V^{\mu }\right)$ is called the *ħ*-quantum big algebra, which interpolates between $G^{\mu }:=G_{1}^{\mu }\subset Q^{\mu }$ the quantum big algebra and $B^{\mu }:=G_{0}^{\mu }\subset C^{\mu }$ the (classical) big algebra.

The universal big algebras ${\left(G_{0}\right)}_{x}$ for x∈s were denoted by $A_{x}\subset U\left(g\right)$ in ref. 10 and its action on a representation *V*^*μ*^ was also studied in *loc. cit.*. Our finite-dimensional matrix algebras $B_{x}^{\mu }$ from Eq. **1.2** are just the images of $A_{x}$ in $\text{End}(V^{\mu })$. Using their results we can deduce the following:

Theorem 2.1. *Let* $\mu \in \Lambda^{+}\left(\text{G}\right)$ *be a dominant character. Then,*

1. *the *ħ*-quantum big algebra* $G_{\hbar }^{\mu }\subset Q_{\hbar }^{\mu }$ *is a maximal commutative subalgebra, finite-free over* $Z_{\hbar }\left(g\right)$, *consequently it contains the ħ-*quantum medium algebra $M_{\hbar }^{\mu }:=Z\left(Q_{\hbar }^{\mu }\right)\subset G_{\hbar }^{\mu }$,
2. *the big algebra* $B^{\mu }=G_{0}^{\mu }\subset C^{\mu }$ *is a maximal commutative subalgebra, finite-free over* $S^{*}{\left(g\right)}^{\text{G}}$, *consequently, the medium algebra* $M^{\mu }≅M_{0}^{\mu }≅Z\left(C^{\mu }\right)\subset B^{\mu }$,
3. *the Hilbert series of* $B^{\mu }$ *satisfies*
  $$\sum_{i=0}^{\infty }\dim\left({\left(B^{\mu }\right)}^{i}\right)q^{i}=\frac{\prod_{\alpha \in \Delta^{+}}\frac{\left(1-q^{\left(\rho +\mu ,\alpha \right)}\right)}{\left(1-q^{\left(\rho ,\alpha \right)}\right)}}{\prod_{j=1}^{r}\left(1-q^{d_{j}}\right)},$$
4. *for all* x∈s *the algebra* $B_{x}^{\mu }\subset \text{End}\left(V^{\mu }\right)$ *acts both with 1-dimensional common eigenspaces and cyclically.*

It was already observed in ref. 10 that *Theorem* 2.1.4 implies that the cyclic action of $B_{e}^{\mu }$ on *V*^*μ*^ endows *V*^*μ*^ with a graded ring structure. The whole big algebra $B^{\mu }$ however contains much more information. For example it follows from *Theorem* 1.2.1 that $B_{h_{z}}^{\mu }$ leaves $z\cdot V_{\lambda }^{\mu }$, the common eigenspaces of $M_{h_{z}}^{\mu }=\varrho \left(U\left(g_{h_{z}}\right)\right)\subset \text{End}{\left(V^{\mu }\right)}^{{\text{G}}_{h_{z}}}$, invariant. Thus by *Theorem* 1.2.2 $B_{e}^{\mu }$ leaves $\lim_{e}V_{\lambda }^{\mu }$ invariant and so we can define the multiplicity algebra

[2.2] $$\begin{array}{c} Q_{\lambda }^{\mu }:=B_{e}^{\mu }{|}_{\lim_{e}V_{\lambda }^{\mu }}\subset \text{End}\left(\lim_{e}V_{\lambda }^{\mu }\right). \end{array}$$

Then *Theorem* 2.1.4 and *Theorem* 1.2 imply the following:

Corollary 2.2. *Let* $\lambda \leq \mu \in \Lambda^{+}\left(\text{G}\right)$ *be dominant characters. The big algebra* $B_{e}^{\mu }$ *at* e∈s *induces Eq.* ***2.2*** *a graded algebra structure* $Q_{\lambda }^{\mu }$ *on* ${\left(\lim_{e}V_{\lambda }^{\mu }\right)}^{*}$ *such that*

1. $\sum \dim{\left(Q_{\lambda }^{\mu }\right)}^{i}q^{(\mu -\lambda ,\rho )-i}=m_{\lambda }^{\mu }\left(q\right)$ *Lusztig’s *q*-analogue of multiplicity Eq.* ***1.8****,*
2. *there are natural quotient maps* $B_{e}^{\mu }↠Q_{\mu_{\min}}^{\mu }↠Q_{\lambda }^{\mu }$*,*
3. $Q_{\mu_{\min}}^{\mu }≅B_{e}^{\mu }/\left({\left(M_{e}^{\mu }\right)}_{+}\right)=B_{e}^{\mu }/\left({\left(M_{1}\right)}_{e},\cdots ,{\left(M_{r}\right)}_{e}\right)$.

### 2.1. Computing Big Algebras.

Fix a basis $\left\{X_{i}\right\}$ for g and a dual basis $\{X^{i}\}\subset g$ with respect to the Killing form of g. For $A\in C^{\mu }$, following Kirillov (1), Wei (11) introduced the following *D*-operator:

$$D\left(A\right):=\frac{1}{2}\sum_{i}\rho_{\mu }\left(X^{i}\right)\frac{\partial (A)}{\partial X_{i}}.$$

It is shown in ref. 11 that $D\left(A\right)\in C^{\mu }$ and that D(A) is independent of the choice of the basis $\{X_{i}\}\subset g$. This *D*-operator allows us to construct new operators from known ones. For example for $p\in C{\left[g\right]}^{\text{G}}$ we have $D\left(p\right)=M_{dp/2}$ is the medium operator of Eq. **1.5**. It is not true that for any $p\in C{\left[g\right]}^{\text{G}}$ iterated derivatives $D^{k}\left(p\right)$ are still in the big algebra $B^{\mu }$. However, starting with a good generating set of $C{\left[g\right]}^{\text{G}}$ we can explicitly generate the big algebra. Here is such an example in type *A*.

Theorem 2.3. *For* $A\in sl_{n}$ *let* $c_{i}\left(A\right)={\left(-1\right)}^{i}(\det\left(\Lambda^{i}\left(A\right)\right)$ *be the *i*th coefficient of the characteristic polynomial of *A*. Then,* $C{\left[sl_{n}\right]}^{{\text{SL}}_{n}}≅C\left[c_{2},\cdots ,c_{n}\right]$ *and the big operators*

$$B_{i,k-i}=D^{i}\left(c_{k}\right)\in C^{\mu }$$

*generate the big algebra*

$$B^{\mu }=C{\left[sl_{n}\right]}^{{\text{SL}}_{n}}{\left\langle B_{i,k-i}\right\rangle }_{0<i<k\leq n}\subset C^{\mu }.$$

Similar generating sets are known in types B,C,D,G and conjectured to exist in all types (8).

## 3. Geometric Aspects

Let G be a connected semisimple complex Lie group, ${\text{G}}^{\vee }$ its Langlands dual group. Their Lie algebras are g and $g^{\vee }$ and t⊂g and $t^{\vee }\subset g^{\vee }$ are Cartan subalgebras with $t^{*}≅t^{\vee }$ naturally. Identify $g≅g^{*}$ and $t≅t^{*}$ by the Killing form. Then, the Duflo isomorphism (12, Lemme V.1) is

[3.1] $$\begin{array}{c} \delta :=\chi^{-1}{}^{\circ}\psi :Z\left(g\right)\to S^{*}{\left(t\right)}^{\text{W}}≅S^{*}{\left(g\right)}^{\text{G}}, \end{array}$$

where $\chi :S^{*}{\left(g\right)}^{\text{G}}≅C{\left[g\right]}^{\text{G}}\to C{\left[t\right]}^{\text{W}}≅S^{*}{\left(t\right)}^{\text{W}}$ is the Chevalley isomorphism and $\psi :Z\left(g\right)\to S^{*}{\left(t\right)}^{\text{W}}$ is the Harish-Chandra isomorphism. On the Rees constructions Eq. **2.1** this induces

$$\begin{array}{c} \delta_{\hbar }:Z_{\hbar }\left(g\right)≅C\left[\hbar \right]{\left[g\right]}^{\text{G}}≅C{\left[g^{\vee }\times C\right]}^{{\text{G}}^{\vee }\times C^{\times }}. \end{array}$$

The following *Theorem* 3.1 shows that our algebras have natural meanings related to equivariant (intersection) cohomology of affine Schubert varieties. All our cohomologies and intersection cohomologies will be with C-coefficients and G-equivariant (intersection) cohomology will be over $H^{2*}\left(B\text{G}\right)≅C{\left[g\right]}^{\text{G}}=H_{\text{G}}^{2*}$. From results in ref. 13, we can deduce the following:

Theorem 3.1. *Let G be a connected semisimple group and* g *its Lie algebra, with Langlands dual* ${\text{G}}^{\vee }$ *and corresponding affine Grassmannian* $\text{Gr}:={\text{Gr}}_{{\text{G}}^{\vee }}={\text{G}}^{\vee }\left(C\left(\left(z\right)\right)\right)/{\text{G}}^{\vee }\left(C\left[\left[z\right]\right]\right)$. *Let* $\mu \in \Lambda^{+}\left(\text{G}\right)$ *be a dominant character and let* ${\text{Gr}}^{\mu }:=\bar{{\text{G}}^{\vee }\left(C\left[\left[z\right]\right]\right)z^{\mu }}$ *be the corresponding* affine Schubert variety, *with action of* ${\text{G}}^{\vee }\subset {\text{G}}^{\vee }\left(C\left[\left[z\right]\right]\right)$ *from the left and* $C^{\times }$ *through loop rotation on *z*. For* $\lambda \leq \mu \in \Lambda^{+}\left(\text{G}\right)$ *we let* $W_{\lambda }^{\mu }:={\text{G}}_{1}^{\vee }\left(C\left[\left[z^{-1}\right]\right]\right)z^{\lambda }\cap {\text{Gr}}^{\mu }$ *be the* affine Grassmannian slice, *where* ${\text{G}}_{1}^{\vee }\left(C\left[\left[z^{-1}\right]\right]\right)$ *is the kernel of the evaluation map* ${\text{G}}^{\vee }\left(C\left[\left[z^{-1}\right]\right]\right)\to {\text{G}}^{\vee }$ at $z^{-1}=0$. *Then,*

1. $H_{{\text{G}}^{\vee }\times C^{\times }}^{2*}\left({\text{Gr}}^{\mu }\right)≅M_{\hbar }^{\mu }$ *as* $H_{{\text{G}}^{\vee }\times C^{\times }}^{2*}≅C\left[\hbar \right]{\left[g\right]}^{\text{G}}$*-algebras,*
2. ${\text{End}}_{H_{{\text{G}}^{\vee }\times C^{\times }}^{2*}\left({\text{Gr}}^{\mu }\right)}\left(IH_{{\text{G}}^{\vee }\times C^{\times }}^{2*}\left({\text{Gr}}^{\mu }\right)\right)≅Q_{\hbar }^{\mu }$ *as* ${\text{H}}_{{\text{G}}^{\vee }\times C^{\times }}^{2*}≅Z_{\hbar }\left(g\right)$*-algebras,*
3. $IH_{{\text{G}}^{\vee }\times C^{\times }}^{2*}\left({\text{Gr}}^{\mu }\right)≅G_{\hbar }^{\mu }$ *as* $H_{{\text{G}}^{\vee }\times C^{\times }}^{*}\left({\text{Gr}}^{\mu }\right)≅M_{\hbar }^{\mu }$*-modules. In particular,* $G_{\hbar }^{\mu }$ *endows* $IH_{{\text{G}}^{\vee }\times C^{\times }}^{2*}\left({\text{Gr}}^{\mu }\right)$ *with a graded ring structure compatible with the action of* $H_{{\text{G}}^{\vee }\times C^{\times }}^{2*}\left({\text{Gr}}^{\mu }\right)≅M_{\hbar }^{\mu }$,
4. $IH^{2*}\left(W_{\lambda }^{\mu }\right)≅Q_{\lambda }^{\mu }$ *as graded vector spaces, thus* $Q_{\lambda }^{\mu }$ *endows* $IH^{2*}\left(W_{\lambda }^{\mu }\right)$ *with a graded ring structure.*

## 4. Examples–Problems

### 4.1. Minuscule and Weight Multiplicity Free Kirillov Algebras.

When *V*^*μ*^ is weight multiplicity free, for example when *μ* is minuscule, the Kirillov algebras are already commutative (14, *Theorem* 4.1), thus $M_{\hbar }^{\mu }≅G_{\hbar }^{\mu }≅C_{\hbar }^{\mu }$. First, we discuss the classical case of $B^{\mu }=G_{0}^{\mu }$.

For any $\mu \in \Lambda^{+}$ we have the unique closed G-orbit $\text{G}v_{\mu }≅\text{G}/P_{\mu }\subset P\left(V^{\mu }\right)$, a partial flag variety. We can form the big zero scheme $Z^{\mu }:=\cap_{B\in B^{\mu }}Z\left(Y_{B}\right)\subset s\times P\left(V^{\mu }\right)$ as the common zeroes of the vector fields $Y_{B}\in X\left(s\times P\left(V^{\mu }\right)\right)$ induced by the big operators $B\in B^{\mu }$, parameterizing their common eigenvectors. By construction $C\left[Z^{\mu }\right]≅B^{\mu }$. On the other hand we can see that $Z\left(Y_{M_{1}}\right)\cap \text{G}v_{\mu }\subset Z^{\mu }\subset s\times P\left(V^{\mu }\right)$, because for a generic x∈s the scheme $Z\left({\left(Y_{M_{1}}\right)}_{x}\right)\cap \text{G}v_{\mu }$ contains only isolated points of $Z({\left(Y_{M_{1}}\right)}_{x})$. From (15, *Theorem* 1.3), we have that $C\left[Z\left(Y_{M_{1}}\right)\cap \text{G}v_{\mu }\right]≅H_{\text{G}}^{2*}\left(\text{G}v_{\mu }\right)$ and thus we always have a surjective map

[4.1] $$\begin{array}{c} B^{\mu }↠H_{\text{G}}^{2*}\left(\text{G}/P_{\mu }\right). \end{array}$$

The ring homomorphism Eq. **4.1** can be thought of an upgrade of a similar linear map $\hat{f}$ in (16, *Theorem* 1), which was proved (essentially) in ref. 17 to be a surjection. When *μ* is minuscule, the Hilbert series of the two graded rings of Eq. **4.1** agree and we get that $B^{\mu }≅H_{\text{G}}^{2*}\left(\text{G}/P_{\mu }\right).$ This result was deduced by algebraic means in (18, §6).

When we use the *ħ* = 0 specialization of *Theorem* 3.1.1 we get that

[4.2] $$\begin{array}{c} B^{\mu }≅M^{\mu }≅H_{{\text{G}}^{\vee }}^{2*}\left({\text{Gr}}^{\mu }\right)≅H_{{\text{G}}^{\vee }}^{2*}\left({\text{G}}^{\vee }/P_{\mu }^{\vee }\right) \end{array}$$

the equivariant cohomology of the cominuscule flag variety. The two descriptions above then agree because ${\text{H}}_{\text{G}}^{2*}\left(\text{G}/P_{\mu }\right)≅C{\left[t\right]}^{{\text{W}}_{\mu }}≅C{\left[t^{*}\right]}^{{\text{W}}_{\mu }}≅C{\left[t^{\vee }\right]}^{{\text{W}}_{\mu }}≅{\text{H}}_{{\text{G}}^{\vee }}^{2*}\left({\text{G}}^{\vee }/P_{\mu }^{\vee }\right),$ where ${\text{W}}_{\mu }:=Stab\left(\mu \right)\subset \text{W}$ in the Weyl group of G.

Similarly, for *V*^*μ*^ weight multiplicity free (18, *Conjecture* 6) suggests G-invariant subvarieties $X_{\mu }\subset P\left(V^{\mu }\right)$ such that $B^{\mu }≅H_{\text{G}}^{2*}\left(X_{\mu }\right)$. For example for the weight multiplicity free $\mu =k\omega_{1}\in \Lambda^{+}\left({\text{SL}}_{n}\right)$ we have $X_{\mu }≅S^{k}\left(P^{n-1}\right)$, the *k*th symmetric product with the diagonal action of ${\text{SL}}_{n}$. With a similar technique as above and straightforwardly extending (15, *Theorem* 1.3) to the orbifold $S^{k}\left(P^{n-1}\right)$ we can prove Panyushev’s conjecture:

[4.3] $$\begin{array}{c} B^{k\omega_{1}}\left(sl_{n}\right)≅H_{{\text{SL}}_{n}}^{2*}\left(S^{k}\left(P^{n-1}\right)\right)≅S_{H_{{\text{SL}}_{n}}^{2*}}^{k}\left(H_{{\text{SL}}_{n}}^{2*}\left(P^{n-1}\right)\right). \end{array}$$

Note that $B^{k\omega_{1}}\left(sl_{n}\right)≅H_{{\text{PGL}}_{n}}^{2*}\left({\text{Gr}}^{k\omega_{1}}\right)$ from *Theorem* 3.1.2. The varieties ${\text{Gr}}^{k\omega_{1}}$ are different from $S^{k}\left(P^{n-1}\right)$ for example $S^{k}\left(P^{1}\right)≅P^{k}$ is smooth while ${\text{Gr}}^{k\omega_{1}}\left({\text{PGL}}_{2}\right)$ is singular for *k* > 1. Still they have isomorphic equivariant cohomology rings:

[4.4] $$\begin{array}{c} H_{{\text{SL}}_{2}}^{2*}\left(P^{k}\right)≅B^{k\omega_{1}}\left(sl_{2}\right)≅H_{{\text{PGL}}_{2}}^{2*}\left({\text{Gr}}^{k\omega_{1}}\right). \end{array}$$

For quantum Kirillov algebras *Theorem* 3.1.2 is useful when *μ* is minuscule. In that case the loop rotation action on ${\text{Gr}}^{\mu }$ is trivial, which implies the surprising

Corollary 4.1. *When* $\mu \in \Lambda^{+}\left(\text{G}\right)$ *is minuscule* $C^{\mu }\left(g\right)≅Q^{\mu }\left(g\right)$ *as* $Z\left(g\right)\overset{\delta }{≅}C{\left[g\right]}^{\text{G}}$*-algebras, where *δ* of Eq.* ***3.1*** *is the Duflo isomorphism.*

The isomorphism can be constructed as the combination of the generalized Harish-Chandra isomorphisms in (19, §9), making it the sought-after generalized Duflo isomorphism in this minuscule case.

Applied to the standard representation $Q^{\omega_{1}}\left(sl_{n}\right)≅C^{\omega_{1}}\left(sl_{n}\right)$*Corollary* 4.1 implies that the Capelli identity matches the classical Cayley-Hamilton identity under the Duflo isomorphism, which is (20, *Theorem* 7.1.1). In types *C* and *D* the case of N=2n in (20, *Theorem* 7.1.6) gives $Q^{\omega_{1}}\left(g\right)≅C^{\omega_{1}}\left(g\right)$. Note that in type *B*, the standard representation is not minuscule. Indeed the case of N=2n+1 in (20, *Theorem* 7.1.6) shows that the quantum Capelli identity does not map to the classical Cayley-Hamilton equation, thus $Q^{\omega_{1}}\left(so_{2n+1}\right)≇C^{\omega_{1}}\left(so_{2n+1}\right)$, which is compatible with the nontriviality of the loop rotation on ${\text{Gr}}^{\omega_{1}}\left({\text{SO}}_{2n+1}\right)$.

### 4.2. Visualization of Explicit Examples.

As the big algebras $B^{\mu }$ are commutative and finite-free over the polynomial ring $H_{\text{G}}^{2*}$, they correspond to affine schemes $\mathrm{Spec}(B^{\mu })$ finite flat over the affine space $\mathrm{Spec}(H_{\text{G}}^{2*})$. With the exception of some small rank examples the embedding dimension of $\mathrm{Spec}(B^{\mu })$ (the minimal number of generators of $B^{\mu }$) is larger than three, thus we cannot directly depict them. For visualization purposes, the principal subalgebras obtained by base changing to a principal ${\text{SL}}_{2}\to \text{G}$ subgroup: $B_{{\text{SL}}_{2}}^{\mu }:=B^{\mu }{⊗}_{H_{\text{G}}^{2*}}{\text{H}}_{{\text{SL}}_{2}}^{2*}$ and $M_{{\text{SL}}_{2}}^{\mu }:=M^{\mu }{⊗}_{H_{\text{G}}^{2*}}{\text{H}}_{{\text{SL}}_{2}}^{2*}$ are better behaved. Their spectra $\mathrm{Spec}(B_{{\text{SL}}_{2}}^{\mu })$ and $\mathrm{Spec}(M_{{\text{SL}}_{2}}^{\mu })$, which we call the big and medium skeletons, are curves over the line $\mathrm{Spec}(H_{{\text{SL}}_{2}}^{2*})$. We call $\text{Spec}(B_{h}^{\mu })$ and $\mathrm{Spec}(M_{h}^{\mu })$, the fibers over the principal semisimple element $h\in sl_{2}//{\text{SL}}_{2}≅\mathrm{Spec}\left(H_{{\text{SL}}_{2}}^{2*}\right)$, the big and medium principal spectra. Because of *Theorem* 1.1 one can identify

[4.5] $$\begin{array}{c} \mathrm{Spec}\left(M_{h}^{\mu }\right)≅\mathrm{Spec}\left(V^{\mu }\right)\subset t^{*}, \end{array}$$

where $\mathrm{Spec}(V^{\mu })$ is the reduced scheme of the set of weights in *V*^*μ*^, which appeared in a closely related context in (17, *Theorem* 1.3.2).

#### 4.2.1. Big algebras for SL_2_.

By Eq. **4.4**, we have $B^{n\omega_{1}}\left(sl_{2}\right)≅H_{{\text{SL}}_{2}}^{2*}\left(P^{n}\right)$, which have been computed in (15, §4.4), yielding Eq. **4.6**.

[4.6] $$\begin{array}{c} B^{n\omega_{1}}\left(sl_{2}\right)≅\left\{\begin{array}{cc} C\left[c_{2},M_{1}\right]/(\left(M_{1}^{2}+n^{2}c_{2}\right)\left(M_{1}^{2}+{\left(n-2\right)}^{2}c_{2}\right)\cdots \left(M_{1}^{2}+4c_{2}\right)M_{1}) & \text{for}\;n\;\text{even};\\ C\left[c_{2},M_{1}\right]/(\left(M_{1}^{2}+n^{2}c_{2}\right)\left(M_{1}^{2}+{\left(n-2\right)}^{2}c_{2}\right)\cdots \left(M_{1}^{2}+9c_{2}\right)\left(M_{1}^{2}+c_{2}\right)) & \text{for}\;n\;\text{odd}. \end{array}\right. \end{array}$$

**Fig. 1.**
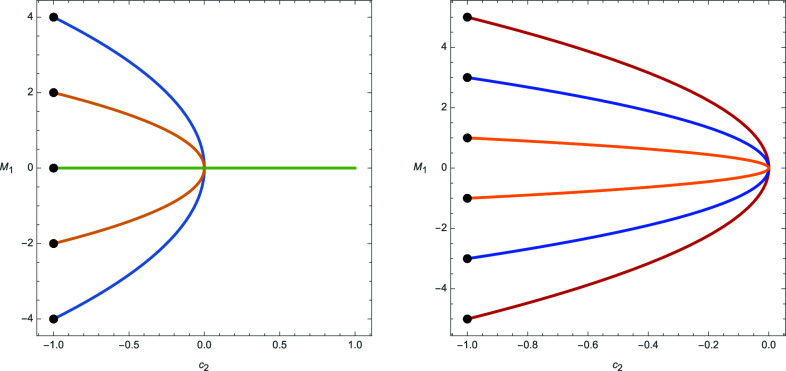
Spec $B^{4\omega_{1}}\left(sl_{2}\right)≅\mathrm{Spec}H_{{\text{SL}}_{2}\left(C\right)}^{*}\left(P^{4}\right)$ and $\mathrm{Spec}B^{5\omega_{1}}\left(sl_{2}\right)≅\mathrm{Spec}H_{{\text{SL}}_{2}\left(C\right)}^{*}\left(P^{5}\right)$.

In Fig. 1, the real points of the spectrum of the big algebras for two ${\text{SL}}_{2}$ examples are shown, with the black dots depicting the principal spectrum, which by Eq. **4.5** can be identified with the weights of the representation.

#### 4.2.2. Big algebra for standard representation of SL_3_.

Using the Cayley-Hamilton identity one can explicitly compute the big algebra for the standard representation of ${\text{SL}}_{3}$ in terms of the small operator *M*_1_ of Eq. **1.4** as

$$B^{\omega_{1}}\left(sl_{3}\right)≅C\left[c_{2},c_{3},M_{1}\right]/\left(M_{1}^{3}+c_{2}M_{1}+c_{3}\right).$$

**Fig. 2.**
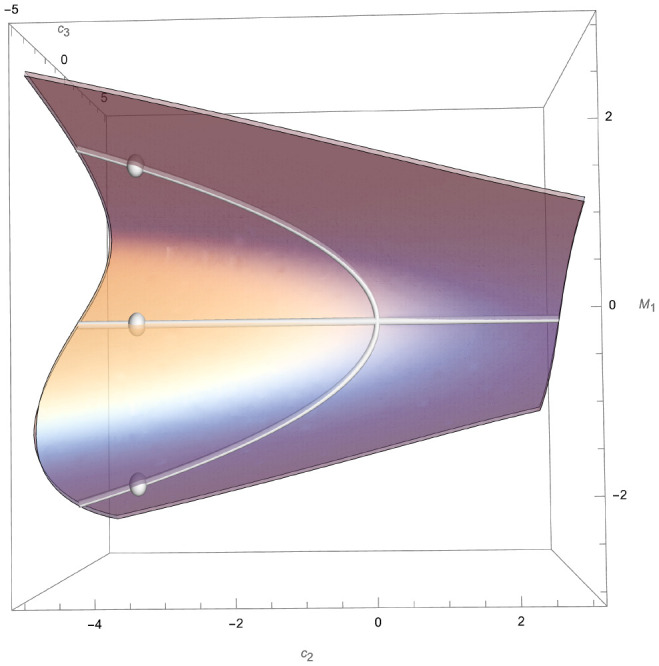
$\mathrm{Spec}B^{\omega_{1}}\left(sl_{3}\right)$, its skeleton $\mathrm{Spec}B_{{\text{SL}}_{2}}^{\omega_{1}}\left(sl_{3}\right)$ and principal spectrum $\mathrm{Spec}B_{h}^{\omega_{1}}\left(sl_{3}\right)$.

Fig. 2 shows the real points of the spectrum of $B^{\omega_{1}}\left(sl_{3}\right)$ together with its skeleton and principal spectrum.

#### 4.2.3. Big algebra for $\rho_{3\omega_{1}}$ of SL_3_—the decuplet.

Using either Eq. **4.3** or *Theorem* 2.3, we can compute the big algebra $B^{3\omega_{1}}\left(sl_{3}\right)≅H_{{\text{SL}}_{3}}^{2*}\left(S^{3}\left(P^{2}\right)\right)$ explicitly in terms of the medium operators $M_{1}=D\left(c_{2}\right)$ and $M_{2}=D\left(c_{3}\right)$:

[4.7] $$\begin{array}{cc} & B^{3\omega_{1}}\left(sl_{3}\right)≅C\left[c_{2},c_{3},M_{1},M_{2}\right]/\\ & \left(\begin{array}{c} M_{1}^{4}-6M_{1}^{2}M_{2}+4M_{1}^{2}c_{2}-18M_{1}c_{3}+3M_{2}^{2}-6M_{2}c_{2},\\ M_{1}^{3}M_{2}+M_{1}^{3}c_{2}+3M_{1}^{2}c_{3}-3M_{1}M_{2}^{2}\\ \;+M_{1}M_{2}c_{2}+4M_{1}c_{2}^{2}-9M_{2}c_{3} \end{array}\right) \end{array}$$

**Fig. 3.**
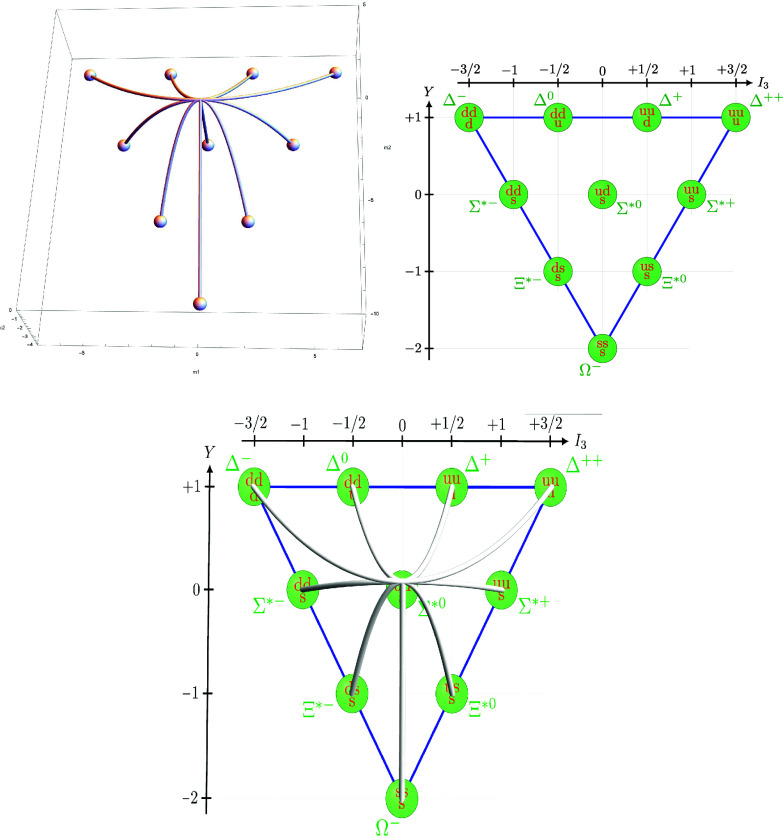
$\mathrm{Spec}(B_{{\text{SL}}_{2}}^{3\omega_{1}}\left(sl_{3}\right))$ over $\mathrm{Spec}(B_{h}^{3\omega_{1}}\left(sl_{3}\right))$, baryon decuplet and skeleton over decuplet.

From this we obtain $B_{{\text{SL}}_{2}}^{3\omega_{1}}$ by setting $c_{3}=0$ and $B_{h}^{3\omega_{1}}$ by further setting $c_{2}=-4$. The first picture of Fig. 3 shows the resulting picture of the real points of the skeleton and the principal spectrum.

The principal spectrum can be identified with the set of weights in $V^{3\omega_{1}}$ by Eq. **4.5**, which in turn corresponds to the particles appearing in the baryon decuplet of Gell-Mann (21, pp. 87, Fig. 1 pp.88); see the second picture in Fig. 3. There are two quantum numbers, the isospin *I*_3_ and hypercharge *Y* which distinguish the particles in the multiplet. They correspond to our operators as ${\left(M_{1}\right)}_{h}=4I_{3}$ and ${\left(M_{2}\right)}_{h}=4Y$. Thus our two relations in our big algebra Eq. **4.7** give the following generating set of polynomial relationships between these two quantum numbers in the baryon decuplet:

[4.8] $$\begin{array}{c} \begin{array}{c} I_{3}\left(Y-1\right)\left(4I_{3}^{2}-3Y-4\right)=0\\ 16I_{3}^{4}-24I_{3}^{2}Y-16I_{3}^{2}+3Y^{2}+6Y=0 \end{array} \end{array}$$

The third picture in Fig. 3 shows that we can obtain the skeleton $\text{Spec}(B_{{\text{SL}}_{2}}^{3\omega_{1}})$ by connecting the particles in the decuplet by parabolas when they correspond to each other under the up–down quark symmetry. The two particles fixed by this symmetry, the $\Sigma^{*0}$ and $\Omega^{-}$, are supporting lines in the skeleton $\mathrm{Spec}(B^{3\omega_{1}}\left(sl_{3}\right))$. $\Omega^{-}$ is the particle formed by three strange quarks, whose existence was famously predicted by Gell-Mann based on this baryon decuplet model (21, pp. 87).

#### 4.2.4. Big algebra of adjoint representation of SL_3_—the octet.

The smallest dimensional nonweight multiplicity free representation is the adjoint representation $\rho_{\omega_{1}+\omega_{2}}$ of ${\text{SL}}_{3}$. In this case $M^{\omega_{1}+\omega_{2}}\left(sl_{3}\right)⊊B^{\omega_{1}+\omega_{2}}\left(sl_{3}\right)$, the medium and big algebras are distinct. Using (14, Table III) or *Theorem* 2.3 one can compute the big algebra, and in turn the medium subalgebra, explicitly, in terms of the medium operators $M_{1}=D\left(c_{2}\right)$ and $M_{2}=D\left(c_{3}\right)$ and big operator $N_{1}=D^{2}\left(c_{3}\right)$:

[4.9] $$\begin{array}{l} B^{\omega_{1}+\omega_{2}}(sl_{3})≅\mathbb{C}[c_{2},c_{3},M_{1},N_{1}]/\\ \;\;\;\left(\begin{array}{c} 3M_{1}^{2}+N_{1}^{2}+12c_{2},\\ M_{1}^{3}N_{1}+c_{2}M_{1}N_{1}-9c_{3}M_{1} \end{array}\right)\\ \;\;\;M^{\omega_{1}+\omega_{2}}(sl_{3})≅\mathbb{C}[c_{2},c_{3},M_{1},M_{2}]/ \end{array}$$

[4.10] $$\left(\begin{array}{l} M_{1}^{2}M_{2}+c_{2}M_{2}+3c_{3}M_{1},\\ M_{1}^{4}+4c_{2}M_{1}^{2}+3M_{2}^{2},\\ 3M_{1}M_{2}^{2}+9c_{3}M_{2}-c_{2}M_{1}^{3}-4c_{2}^{2}M_{1} \end{array}\right)$$

**Fig. 4.**
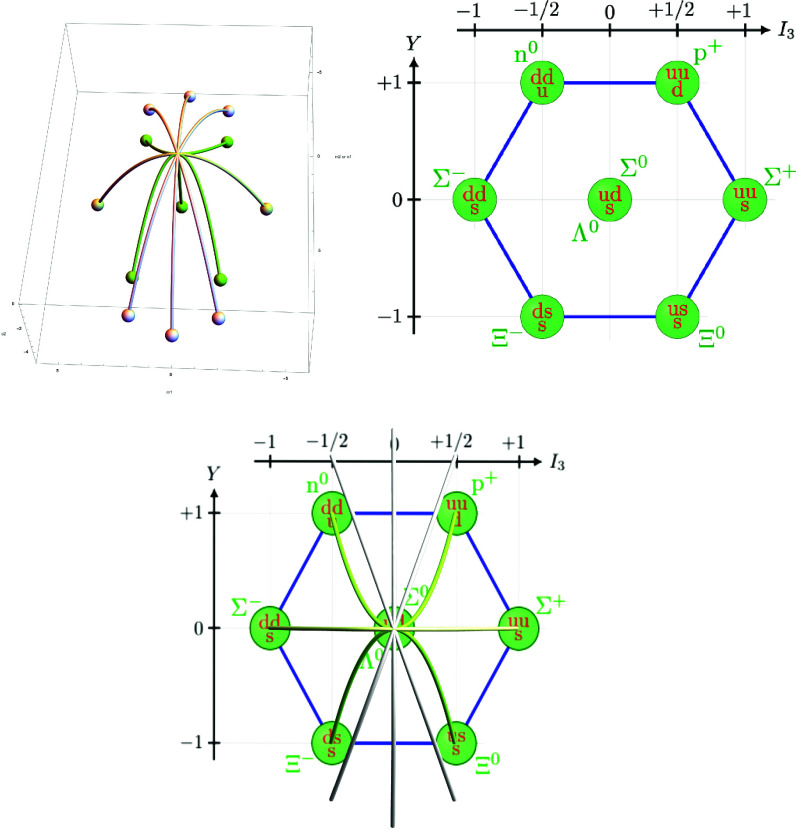
Skeletons $B_{{\text{SL}}_{2}}^{\omega_{1}+\omega_{2}}\left(sl_{3}\right)$, $M_{{\text{SL}}_{2}}^{\omega_{1}+\omega_{2}}\left(sl_{3}\right)$ over $B_{h}^{\omega_{1}+\omega_{2}}\left(sl_{3}\right)$, $M_{h}^{\omega_{1}+\omega_{2}}\left(sl_{3}\right)$, baryon octet and big and medium skeletons over octet.

Setting $c_{3}=0$ in these equations gives us the big and medium skeletons, why further specializing $c_{2}=-4$ gives us the big and medium principal spectra. These are depicted (white for big and green for medium) on the first picture of Fig. 4. We used the coordinates $c_{2},M_{1}$ and *N*_1_ for the big skeleton but $c_{2},M_{1}$ and $M_{2}=\frac{1}{3}M_{1}N_{1}$ for the medium skeleton.

Thus our relations in Eq. **4.10** imply the following generating set of polynomial relations between the quantum numbers *I*_3_ and *Y* in the baryon octet (see second picture in Fig. 4):

[4.11] $$\begin{array}{c} \begin{array}{c} Y\left(2I_{3}-1\right)\left(2I_{3}+1\right)=0\\ 4I_{3}^{3}+3I_{3}Y^{2}-4I_{3}=0\\ 16I_{3}^{4}-16I_{3}^{2}+3Y^{2}=0 \end{array} \end{array}$$

We can also compute the multiplicity algebra of the 0 weight from Eq. **4.9** and *Corollary* 2.2 to get

$$Q_{0}^{\omega_{1}+\omega_{2}}\left(sl_{3}\right)≅B^{\omega_{1}+\omega_{2}}/\left({\left(M^{\omega_{1}+\omega_{2}}\right)}_{+}\right)≅C\left[N_{1}\right]/\left(N_{1}^{2}\right).$$

On the third picture of Fig. 4, we can see that the medium skeleton can be built on the baryon octet by connecting the particles corresponding by up–down quark symmetry—such as the neutron $n^{0}$ and proton $p^{+}$—with parabolas. The big skeleton is more complicated. It consists of four parabolas (one shared with the medium skeleton) and has two points in its principal spectrum over the origin in the baryon octet corresponding to the multiplicity two 0 weight space containing the two particles $\Sigma^{0}$ and $\Lambda^{0}$.

***Remark 4.1:*** Using (22), where the Kirillov algebra is computed for the adjoint representation of any simple complex Lie group, one can work out the generators and relations for the corresponding big algebras explicitly. In particular, one can also compute explicitly $B^{2\omega_{2}}\left(so_{5}\right)\subset C^{2\omega_{2}}\left(so_{5}\right)$ the big algebra of the adjoint representation of ${\text{SO}}_{5}$. We can obtain this adjoint representation by restricting the representation $\rho_{\omega_{2}}$ of ${\text{SL}}_{5}$ to the subgroup ${\text{SO}}_{5}\subset {\text{SL}}_{5}$. This way we also have a commutative subalgebra $B^{\omega_{2}}\left(sl_{5}\right){⊗}_{H_{{\text{SL}}_{5}}^{2*}}H_{{\text{SO}}_{5}}^{2*}\subset C^{2\omega_{2}}\left(so_{5}\right).$ Both subalgebras of $C^{2\omega_{2}}\left(so_{5}\right)$ satisfy properties 2., 3., and 4. in *Theorem* 2.1 but can be shown to be nonisomorphic. This shows that the big algebra $B^{2\omega_{2}}\left(so_{5}\right)\subset C^{2\omega_{2}}\left(so_{5}\right)$ is not uniquely determined by these properties.

### 4.3. Twining Big Algebras.

For a connected semisimple complex Lie group G let σ:G→G be a distinguished automorphism, i.e., one which fixes a pinning. In particular, it is induced from an automorphism, also denoted *σ*, of the Dynkin diagram. Examples for the symmetric pair $\left(\text{G},{\text{G}}^{\sigma }\right)$ are $({\text{SL}}_{2n+1},{\text{SO}}_{2n+1}),$ $({\text{SL}}_{2n},{\text{Sp}}_{n}),$ $({\text{SO}}_{2n},{\text{SO}}_{2n-1}),$ $\left({\mathrm{PSO}}_{8},G_{2}\right)$ or $(E_{6},F_{4}).$ Except for the order three *σ* in the case $\left({\mathrm{PSO}}_{8},G_{2}\right)$ the automorphism *σ* is an involution.

The Dynkin diagram automorphism *σ* induces a distinguished automorphism $\sigma :{\text{G}}^{\vee }\to {\text{G}}^{\vee }$ of the Langlands dual. Define the endoscopy group ${\text{G}}_{\sigma }={\left({\left({\text{G}}^{\vee }\right)}_{0}^{\sigma }\right)}^{\vee }$. Such a *σ* will induce an automorphism of the Feigin–Frenkel center, the Gaudin algebra and the universal big algebra, and in turn for $\mu \in \Lambda^{+}{\left(\text{G}\right)}^{\sigma }$ on the big algebra $\sigma :B^{\mu }\to B^{\mu }$. Decompose $B^{\mu }={⊕}_{\kappa \in \hat{\left\langle \sigma \right\rangle }}{\left(B^{\mu }\right)}_{\kappa }$ according to characters of the cyclic group ⟨σ⟩⊂Aut(G). Define the coinvariant algebra $B_{\sigma }^{\mu }:=B^{\mu }/\left({⊕}_{1\neq \kappa \in \hat{\left\langle \sigma \right\rangle }}{\left(B^{\mu }\right)}_{\kappa }\right)$, which computes the ring of functions of the fixed point scheme: $B_{\sigma }^{\mu }≅C\left[\mathrm{Spec}{\left(B^{\mu }\right)}^{\sigma }\right]$. We have the following^*^.

Conjecture 4.1. *For* $\mu \in \Lambda^{+}\left({\text{G}}_{\sigma }\right)$ *also denote the corresponding dominant weight by* $\mu \in \Lambda^{+}{\left(\text{G}\right)}^{\sigma }$. *Then,*

[4.12] $$\begin{array}{c} B_{\sigma }^{\mu }\left(g\right)≅B^{\mu }\left(g_{\sigma }\right). \end{array}$$

The main motivation for the conjecture was that it is compatible with Jantzen’s twining character formula. Namely take $\lambda \in \Lambda^{+}\left({\text{G}}_{\sigma }\right)$ and the corresponding $\lambda \in \Lambda^{+}{\left(\text{G}\right)}^{\sigma }$. The weight space $V_{\lambda }^{\mu }\left(\text{G}\right)$ of the G-representation will inherit an action $\sigma :V_{\lambda }^{\mu }\left(\text{G}\right)\to V_{\lambda }^{\mu }\left(\text{G}\right)$, which combined with the induced action in the big algebra $\sigma :B^{\mu }\to B^{\mu }$ will yield an automorphism of the multiplicity algebra $Q_{\lambda }^{\mu }\left(g\right)$. Then, we expect Eq. **4.12** implies that $Q_{\lambda }^{\mu }{\left(g\right)}_{\sigma }=Q_{\lambda }^{\mu }\left(g_{\sigma }\right)$ and $\dim\left(Q_{\lambda }^{\mu }{\left(g\right)}_{\sigma }\right)=\text{tr}\left(\sigma :Q_{\lambda }^{\mu }\left(g\right)\to Q_{\lambda }^{\mu }\left(g\right)\right)$, when the trace is nonzero. In this case, we get that $\text{tr}\left(\sigma :V_{\lambda }^{\mu }\left(\text{G}\right)\to V_{\lambda }^{\mu }\left(\text{G}\right)\right)=\text{tr}\left(\sigma :Q_{\lambda }^{\mu }\left(g\right)\to Q_{\lambda }^{\mu }\left(g\right)\right)=\;\dim\left(Q_{\lambda }^{\mu }\left(g_{\sigma }\right)\right)=\;\dim\left(V_{\lambda }^{\mu }\left({\text{G}}_{\sigma }\right)\right)$, which is Jantzen’s twining formula (24, Satz 9).

Geometrically the result should follow from the induced action $\sigma :{\text{Gr}}^{\mu }\left({\text{G}}^{\vee }\right)\to {\text{Gr}}^{\mu }\left({\text{G}}^{\vee }\right)$ for $\mu \in \Lambda^{+}{\left(\text{G}\right)}^{\sigma }$. In fact, the first check on the conjecture is when $V^{\mu }\left(\text{G}\right)$ is a *σ*-invariant minuscule representation. When $\mu =\omega_{n}\in \Lambda^{+}\left({\text{SL}}_{2n}\right)$ then σ(μ)=μ and the corresponding cominuscule flag variety ${\text{Gr}}^{n\omega_{1}}\left({\text{PGL}}_{2n}\right)≅\text{Gr}\left(n,C^{2n}\right)$ is the Grassmannian of *n*-planes in $C^{2n}$. The action of *σ* on $\text{Gr}(n,C^{2n})$ is given by σ(V):=ann(ω(V)), where $\omega :C^{2n}\to {\left(C^{2n}\right)}^{*}$ is a symplectic form. Thus we see that $\text{Gr}{\left(n,C^{2n}\right)}^{\sigma }≅L\text{Gr}\left(n,C^{2n}\right)≅{\text{Gr}}^{\omega_{n}}\left({\text{PSp}}_{2n}\right)$ is the Lagrangian Grassmannian. As $\text{Gr}(n,C^{2n})$ is ${\text{PGL}}_{2n}$-regular and $L\text{Gr}(n,C^{2n})$ is ${\text{PSp}}_{2n}$-regular, from (15, *Theorem* 1.3), we can deduce that $B^{\omega_{n}}{\left(sl_{2n}\right)}_{\sigma }≅H_{{\text{PGL}}_{2n}}^{2*}{\left(\text{Gr}\left(n,C^{2n}\right)\right)}_{\sigma }≅H_{{\text{PGL}}_{2n}^{\sigma }}^{2*}\left(\text{Gr}{\left(n,C^{2n}\right)}^{\sigma }\right)≅H_{{\text{PSp}}_{2n}}^{2*}\left(L\text{Gr}\left(n,C^{2n}\right)\right)≅B^{\omega_{n}}\left(so_{2n+1}\right).$

Finally we note that in the example $\text{G}={\text{PGL}}_{3}$ and ${\text{G}}_{\sigma }={\text{SL}}_{2}$ the weight $\omega_{1}\in \Lambda^{+}\left({\text{SL}}_{2}\right)$ corresponds to $\omega_{1}+\omega_{2}\in \Lambda^{+}{\left({\text{PGL}}_{3}\right)}^{\sigma }$. Then, we have $\sigma \left(M_{1}\right)=M_{1}$, $\sigma \left(N_{1}\right)=-N_{1}$ and $\sigma \left(M_{2}\right)=-M_{2}$ and so the corresponding $B^{\omega_{1}+\omega_{2}}{\left(sl_{3}\right)}_{\sigma }≅B^{\omega_{1}}\left(sl_{2}\right)$ can be seen in the first picture of Fig. 4. Namely, the fixed point scheme of *σ* on $\mathrm{Spec}(B^{\omega_{1}+\omega_{2}}\left(sl_{3}\right))$ is the common parabola of the big skeleton shared with the medium skeleton, where $N_{1}=M_{2}=0$.

### 4.4. Mirror Symmetry and Big Spectral Curves.

Big algebras first appeared in ref. 25 in connection with mirror symmetry (26, 27). They were needed to endow the universal G-Higgs bundle in an irreducible representation with the structure of a bundle of algebras along the Hitchin section. Turning the logic back, one can use the big algebras $B^{\mu }$ to define a bundle of algebras on the G-Higgs bundle in the irreducible representation *V*^*μ*^ along the Hitchin section, yielding *big spectral curves*$C^{\mu }\subset {⊕}_{k=1}^{rank(\text{G})}{⊕}_{0<i<k}K^{d_{k}-i}$ living in the total space of direct sum of line bundles *K*^*i*^ for each degree *i* generator of the big algebra. In turn, for any G-Higgs bundle one can construct a big algebra of big Higgs fields in any irreducible representation *V*^*μ*^, which will yield a rank 1 sheaf on the corresponding big spectral curve *C*^*μ*^. We expect a full theory of BNR correspondences for each big spectral curve, bridging the usual spectral curves in ref. 28 with the cameral covers in ref. 29.

Finally, we expect that the geometric description of the quantum big algebras $G^{\mu }$ in ref. 30 as rings of functions on certain spaces of opers, and the description (25) of the big algebras $B^{\mu }$ as rings of functions on upward flows in the Hitchin system could be unified as a description of the *ħ*-quantum big algebras $G_{\hbar }^{\mu }$ on upward flows in $M_{\mathrm{Hodge}}$, the moduli space of *ħ*-connections.

Details of the proofs of the results in this paper, and detailed study of the examples mentioned above will appear elsewhere.

## Data Availability

There are no data underlying this work.

## Footnotes

^*^A proof of this conjecture appeared in ref. 23.

## References

[1] A. A. Kirillov, Family algebras. Electron. Res. Announc. Amer. Math. Soc. **6**, 7–20 (2000).

[2] B. Kostant, Fomenko-Mischenko theory, Hessenberg varieties, and polarizations. Lett. Math. Phys. **90**, 253–285 (2009).

[3] B. Kostant, Lie group representations on polynomial rings. Amer. J. Math. **85**, 327–404 (1963).

[4] R. K. Brylinski, Limits of weight spaces, Lusztig’s *q*-analogs, and fiberings of adjoint orbits. J. Am. Math. Soc. **2**, 517–533 (1989).

[5] G. Lusztig, “Singularities, character formulas, and a *q*-analog of weight multiplicities” in *Analysis and Topology on Singular Spaces, II, III (Luminy, 1981)*, Astérisque (Soc. Math. France, Paris, 1983), vol. 101–102, pp. 208–229.

[6] B. Feigin, E. Frenkel, N. Reshetikhin, Gaudin model, Bethe ansatz and critical level. Commun. Math. Phys. **166**, 27–62 (1994).

[7] L. G. Rybnikov, The shift of invariants method and the Gaudin model. Funct. Anal. Appl. **40**, 188–199 (2006).

[8] O. Yakimova, Symmetrisation and the Feigin-Frenkel centre. Compos. Math. **158**, 585–622 (2022).

[9] B. Feigin, E. Frenkel, “Affine Kac-Moody algebras at the critical level and Gelfand-Dikii algebras” in *Infinite Analysis, Part A, B (Kyoto, 1991)*, Adv. Ser. Math. Phys. (World Sci. Publ., River Edge, NJ, 1992), vol. 16, pp. 197–215.

[10] B. Feigin, E. Frenkel, L. Rybnikov, Opers with irregular singularity and spectra of the shift of argument subalgebra. Duke Math. J. **155**, 337–363 (2010).

[11] Z. Wei, The noncommutative Poisson bracket and the deformation of the family algebras. J. Math. Phys. **56**, 071703 (2015).

[12] M. Duflo, Caractères des groupes et des algèbres de Lie résolubles. Ann. Sci. École Norm. Sup. **4**, 23–74 (1970).

[13] R. Bezrukavnikov, M. Finkelberg, Equivariant Satake category and Kostant-Whittaker reduction. Mosc. Math. J. **8**, 39–72 (2008).

[14] N. Rozhkovskaya, Commutativity of quantum family algebras. Lett. Math. Phys. **63**, 87–103 (2003).

[15] T. Hausel, K. Rychlewicz, Spectrum of equivariant cohomology as fixed point scheme. arXiv [Preprint] (2022). http://arxiv.org/abs/2212.11836 (Accessed 21 August 2024).

[16] R. Friedman, J. W. Morgan, “Minuscule representations, invariant polynomials, and spectral covers” in *Vector Bundles and Representation Theory (Columbia, MO, 2002)*, Contemp. Math. (Amer. Math. Soc., Providence, RI, 2003), vol. 322, pp. 1–41.

[17] V. Ginzburg, Variations on themes of Kostant. Transform. Groups **13**, 557–573 (2008).

[18] D. I. Panyushev, Weight multiplicity free representations, g-endomorphism algebras, and Dynkin polynomials. J. Lond. Math. Soc. **2**, 273–290 (2004).

[19] N. Higson, “On the analogy between complex semisimple groups and their Cartan motion groups” in *Noncommutative Geometry and Global Analysis*, Contemp. Math. (Amer. Math. Soc., Providence, RI, 2011), vol. 546, pp. 137–170.

[20] A. Molev, *Yangians and classical Lie algebras, Mathematical Surveys and Monographs* (American Mathematical Society, Providence, RI, 2007), vol. 143, p. pp. xviii+400.

[21] M. Gell-Mann, Y. Ne’eman, *The Eightfold Way: A Review with A Collection of Reprints* (W.A. Benjamin, Inc., 1964).

[22] M. Tai, “Family algebras and the isotypic components of g tensor g,” PhD thesis, University of Pennsylvania, ProQuest LLC, Ann Arbor, MI (2014), p. 118.

[23] V. Zveryk, Dynkin automorphism actions on Gaudin algebras. arXiv [Preprint] (2023). http://arxiv.org/abs/2311.11872 (Accessed 21 August 2024).

[24] J. C. Jantzen, Darstellungen halbeinfacher algebraischer Gruppen und zugeordnete kontravariante Formen. *Bonn. Math. Schr.* **67**, 124S. (1973).

[25] T. Hausel, “Mirror symmetry and big algebras” in *Minicourse at Conference on Mirror Symmetry Langlands Duality and Hitchin System* (ICMAT, Madrid, 2023). https://hausel.ist.ac.at/videos-and-slides-of-minicourse-in-madrid-available/.

[26] T. Hausel, N. J. Hitchin, Very stable Higgs bundles, equivariant multiplicity and mirror symmetry. Invent. math. **228**, 893–989 (2022).

[27] T. Hausel, “Enhanced mirror symmetry for langlands dual Hitchin systems” in *ICM—International Congress of Mathematicians*, D. Beliaev, S. Smirnov, Eds. (EMS Press, Berlin, 2023), vol. 3, pp. 2228–2249, sec. 1–4.

[28] N. J. Hitchin, Stable bundles and integrable systems. Duke Math. J. **54**, 91–114 (1987).

[29] R. Donagi, “Spectral covers” in *Current Topics in Complex Algebraic Geometry (Berkeley, CA, 1992/93)*, Math. Sci. Res. Inst. Publ. (Cambridge Univ. Press, Cambridge, 1995), vol. 28, pp. 65–86.

[30] B. Feigin, E. Frenkel, V. Toledano Laredo, Gaudin models with irregular singularities. Adv. Math. **223**, 873–948 (2010).

